# Supplementary material for: Characterizing cell-type spatial relationships across length scales in spatially resolved omics data
Source: Nat Commun. 2025 Jan 3;16:350. doi: 10.1038/s41467-024-55700-1 (PMC11699133; doi:10.1038/s41467-024-55700-1)
Supplement: Supplementary file 3 — Supplementary Software 1 [file 41467_2024_55700_MOESM3_ESM.zip › CRAWDAD/plots/seqfish/S5_seqfish_latMeso_cooccurance.pdf]

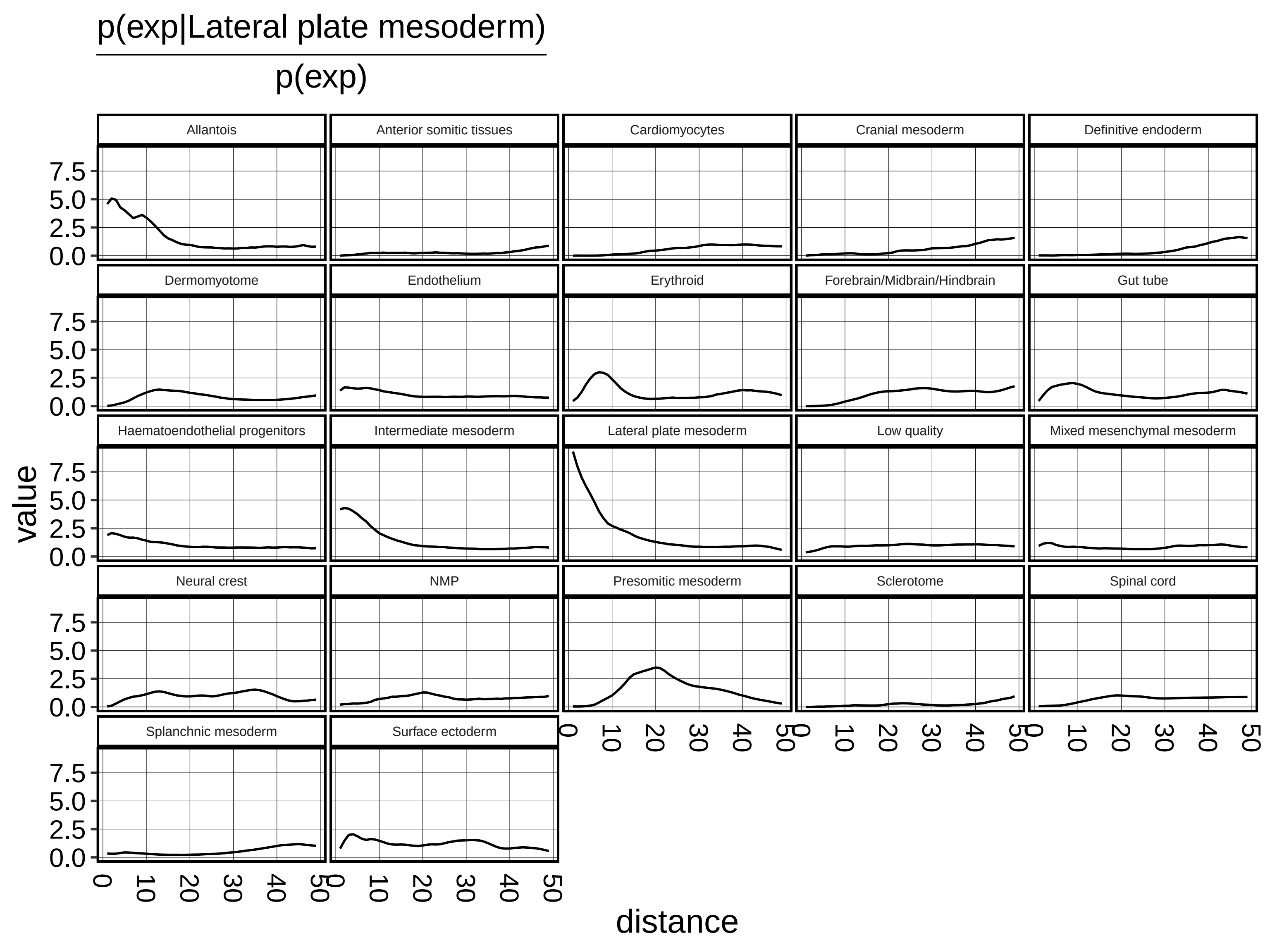
p(exp|Lateral plate mesoderm) / p(exp)
Allantois
Anterior somitic tissues
Cardiomyocytes
Cranial mesoderm
Definitive endoderm
Dermomyotome
Endothelium
Erythroid
Forebrain/Midbrain/Hindbrain
Gut tube
Haematoendothelial progenitors
Intermediate mesoderm
Lateral plate mesoderm
Low quality
Mixed mesenchymal mesoderm
Neural crest
NMP
Presomitic mesoderm
Sclerotome
Spinal cord
Splanchnic mesoderm
Surface ectoderm
value
0.0
2.5
5.0
7.5
0
10
20
30
40
50
distance
